# Relationship between clozapine exposure and the onset of appendicitis in schizophrenia patients: a retrospective cohort study

**DOI:** 10.1186/s12888-022-04312-4

**Published:** 2022-10-21

**Authors:** Yuta Kawakita, Masahiro Takeshima, Tomonari Komatsu, Aya Imanishi, Dai Fujiwara, Yu Itoh, Kazuo Mishima

**Affiliations:** 1 Department of Neuropsychiatry, Akita City Hospital, 4-30 Kawamoto Matsuokamachi, Akita City, 010-0933 Japan; 2 Department of Neuropsychiatry, Akita University Graduate School of Medicine, Akita, Japan; 3 Department of Neuropsychiatry, Noshiro Kousei Medical Center, Akita, Japan

**Keywords:** Clozapine, Appendicitis, Antipsychotics, Schizophrenia

## Abstract

**Objective:**

Clozapine may cause serious side effects despite benefits in patients with schizophrenia. Thus, an accurate understanding of the side-effect profile of clozapine is extremely important in the management of its administration to patients with schizophrenia. Our aim was to validate the relationship between clozapine exposure and appendicitis onset in patients with schizophrenia.

**Methods:**

In this study, we retrospectively compared the incidence and cumulative incidence of appendicitis in patients with schizophrenia with and without a history of clozapine exposure. Among the patients with schizophrenia who visited our hospital between June 2009 and August 2021, we extracted those with a history of clozapine treatment. Patients with a history of taking clozapine were defined as the clozapine exposure group, while the others were defined as the clozapine non-exposure group. Patients with a history of appendectomy before their initial visit to our hospital or with a history of clozapine use at other hospitals were excluded.

**Results:**

There were 65 patients in the clozapine exposure group and 400 patients in the clozapine non-exposure group who met the inclusion criteria. The exposure group exhibited a remarkably higher incidence of appendicitis during the observation period than the non-exposure group (863 cases vs. 124 cases per 100,000 person-years). In particular, if limited to the period of clozapine exposure, the incidence of appendicitis is extremely high, at 2,086 cases per 100,000 person-years. Moreover, multivariable analysis showed that clozapine exposure was an independent factor contributing to the onset of appendicitis.

**Conclusions:**

Clozapine exposure is associated with appendicitis onset in patients with schizophrenia.

## Background

Schizophrenia is a severe psychotic disorder that presents with positive, negative, and cognitive symptoms [1]. Treatment-resistant schizophrenia (TRS) has been clinically defined as the persistence of symptoms despite at least two trials of antipsychotic drugs of adequate dose and duration and occurs in approximately one-third of all patients with schizophrenia [2, 3].

Clozapine is recognized as the “gold standard” drug for the management of TRS and is the only antipsychotic drug approved for TRS [4, 5]. Clozapine has widely accepted clinical benefits for TRS; however, it may cause serious side effects such as myocarditis, granulocytopenia, ileus, and glucose intolerance [4, 5]. Thus, an accurate understanding of the side-effect profile of clozapine is extremely important in the management of its administration to patients with TRS.

Incidentally, individuals with severe mental illnesses, represented by schizophrenia, have a higher prevalence of comorbidities, such as obesity, hypertension, diabetes mellitus, and metabolic syndrome [6]. Appendicitis is one of the most common abdominal emergencies and one of the most frequent indications for emergency surgery [7]. The approximate incidence of appendicitis is 90 − 110 cases per 100,000 persons per year, although it varies slightly between countries, regions, races, and ages [7–12]. Appendicitis occurs frequently from adolescence to thirties, with the incidence peaking in teens and gradually declining over the years [7–12]. Appendicitis often causes peritonitis or abdominal abscess if prompt diagnosis and appropriate treatment are not undergone [7–9]. Meanwhile, patients with schizophrenia are known to often exhibit less pain sensitivity, called “analgesia,” which has been described by Kraepelin and Bleuler since the early twentieth century [13, 14]. It has been reported that if a patient with schizophrenia develops appendicitis, the awareness and detection of abdominal symptoms are delayed owing to less pain sensitivity, which often leads to more severe conditions such as perforated appendicitis [15–17]. Regarding the association between clozapine use and the onset of appendicitis, a single-center retrospective observational study in Germany found that the incidence of appendicitis was remarkably high (2,726 cases per 100,000 person-years) in patients with schizophrenia treated with clozapine [18]. To the best of our knowledge, this study is the only one that referred to this relationship; however, it did not adequately provide a statistical verification of whether clozapine exposure is a risk factor for appendicitis onset among patients with schizophrenia. Verifying the relationship between clozapine exposure and the onset of appendicitis and identifying risk factors for developing appendicitis during clozapine administration will enable the detection of patients at high risk of appendicitis, which will allow psychiatrists to more safely manage patients taking clozapine.

Therefore, the aim of this study was to validate the relationship between clozapine exposure and the onset of appendicitis and to identify risk factors for the onset of appendicitis in patients with schizophrenia, comparing the incidence and cumulative incidence of appendicitis between schizophrenia patients with and without a history of clozapine exposure.

## Methods

### Study design and participants

This retrospective cohort study was approved by the ethics committee of Akita University (approval number: 2762). In this study, we compared the incidence and cumulative incidence of appendicitis in schizophrenia patients with and without a history of clozapine exposure. Among the patients with schizophrenia who visited our hospital between June 2009 and August 2021, we extracted those with a history of clozapine treatment at our hospital. Patients with a history of taking clozapine were defined as the clozapine exposure group, and the others were defined as the clozapine non-exposure group. Patients with a history of appendectomy before their initial visit to our hospital or with a history of clozapine use at other hospitals were excluded. Moreover, patients whose accuracy of their statements on medical history was questionable due to their schizophrenic medical condition and the absence of reliable relatives were excluded from this study. The diagnosis of schizophrenia was confirmed according to the DSM-IV text revision or DSM-5 criteria [19, 20]. The observation period was from the date of the first visit to our hospital until August 2021. The endpoint in this study was defined as the onset of appendicitis during the observation period, and dropout was defined as the discontinuation of follow-up due to transfer to other hospitals or interruption of hospital visits at the patient’s discretion.

Clozapine administration was limited to patients with TRS who exhibited poor response and tolerance criteria. A poor response was defined as failure to respond for a sufficient period (at least 4 weeks) of treatment with a sufficient dose of at least two well-tolerated antipsychotics (including at least one atypical antipsychotic, such as risperidone, perospirone, olanzapine, quetiapine, or aripiprazole, at an equivalent dose of over 600 mg/day chlorpromazine). Poor tolerance was defined as the failure to adequately respond to monotherapy with at least two atypical antipsychotics due to failure to increase the dose to a necessary level for any of the following reasons: occurrence or worsening of moderate or more severe tardive dyskinesia, tardive dystonia or other tardive extrapyramidal symptoms, occurrence of uncontrolled parkinsonian symptoms, akathisia, or acute dystonia [21]. As previously described, the clozapine dose was slowly increased to achieve the optimal dose (the maximum dose in Japan is 600 mg/day) [22]. In consideration of the established discontinuation criteria of the Japanese Guideline Pharmacological Therapy of Schizophrenia and Japanese Clozaril® Patient Monitoring Service, clozapine administration was discontinued due reasons such as hematological adverse events (neutropenia and leukopenia), cardiomyopathy, patient refusal due to drowsiness [21]. Patients who discontinued clozapine immediately resumed treatment with other antipsychotics and remained under observation until the final follow-up date. The diagnosis of appendicitis was confirmed by appendectomy and histopathological evaluation, except in one case. In one of the patients in the clozapine non-exposure group, appendicitis was diagnosed using computed tomography and treated with non-operative management.

### Survey methods

We retrospectively extracted the following demographic and clinical data from electronic medical records: age at the start of the observation, sex, date of developing appendicitis, date of diagnosis of schizophrenia, date of transfer to other hospitals, prescription status of clozapine, other antipsychotics, and the prescription status of concomitant drugs such as anticholinergic drugs, antidepressants (noradrenergic and specific serotonergic antidepressants (NaSSA), selective serotonin reuptake inhibitors (SSRIs), serotonin norepinephrine reuptake inhibitors (SNRIs), and tricyclic antidepressants (TCA)), benzodiazepines, corticosteroids, laxatives, mood stabilizers, and non-steroidal anti-inflammatory drugs (NSAIDs).

The incidence and cumulative incidence of appendicitis during clozapine administration were calculated in the clozapine exposure group, and those during the observation period from the initial visit until the final date of follow-up were also calculated in both groups. Moreover, multivariate analyses were used to examine the risk factors for the onset of appendicitis.

### Statistical analysis

Values are expressed as the median (interquartile range). Differences between the two groups were analyzed using the Wilcoxon rank sum test for continuous variables and the Pearson χ^2^ test or Fisher’s exact probability test for categorical variables. The event-free survival length was determined from the date at the start of the observation or clozapine administration to the onset date of appendicitis or the final date of follow-up. Cumulative incidence curves were derived using the Kaplan–Meier method, and differences between the curves were analyzed using the log-rank test. Odds ratios for developing appendicitis were assessed using multiple logistic regression models. Variables added to the multivariate analysis were determined using backward-elimination stepwise regression. Model selection was based on the minimum Akaike information criterion (AIC). In the logistic regression, we utilized a likelihood ratio χ^2^ test and McFadden’s pseudo R^2^ for the goodness of fit measures. Statistical analyses were performed using JMP 14.3 (SAS Institute, Cary, NC, USA). Statistical significance values of *p* < 0.05 (two-sided) were considered significant.

## Results

### Patient characteristics of the clozapine exposure and non-exposure groups

Among the 492 patients with schizophrenia who visited our hospital between June 2009 and August 2021, 65 patients in the clozapine exposure group and 400 patients in the clozapine non-exposure groups. The characteristics of all the patients in both groups are summarized in Table 1. All appendicitis cases in the clozapine exposure group were observed during clozapine administration. Moreover, the number of appendicitis cases was significantly higher in the clozapine exposure group than in the clozapine non-exposure group [5 (7.7%) vs. 5 (1.3%), *p* = 0.007). Correspondingly, the clozapine exposure group exhibited a remarkably higher incidence of appendicitis during the observation period than the clozapine non-exposure group (863 cases per 100,000 person-years vs. 124 cases per 100,000 person-years). In particular, if limited to the period of clozapine administration, the incidence of appendicitis is extremely high at 2,086 cases per 100,000 person-years.

**Table 1:** Patient characteristics of the clozapine exposure and non-exposure groups

**Characteristics**	**CLZ**	**non-CLZ**	***p*-value**
	*N* = 65	*N* = 400	
Sex, Man	21 (32.3%)	171 (42.7%)	.135
Number of appendicitis cases	5 (7.7%)	5 (1.3%)	**.007**
Incidence of appendicitis during the observation period, (per 1,00,000 person-years)	863	124	-
Incidence of appendicitis during clozapine administration (per 1,00,000 person-years)	2086	-	-
Age at the start of clozapine administration	32.3 (23.1–50.4)	-	-
Length of disease duration at the start of clozapine administration (years)	9.3 (2.9–16.6)	-	-
Duration of clozapine administration (months)	41.5 (8.6–73.7)	-	-
Withdrawal of clozapine administration due to adverse events	18 (27.7%)	-	-
Maximum administration dose of clozapine (mg)	300 (200–525)	-	-
Age at the start of treatment for schizophrenia	20.5 (18.5–26.5)	25.2 (19.8–32.7)	** < .001**
Age at the first visit to our hospital	25.8 (19.9–35.3)	30.1 (23.0–40.9)	**.008**
Length of disease duration (years)	13.3 (7.3–20.8)	12.3 (4.7–23.3)	.665
Length of the observation period (years)	7.4 (2.6–13.1)	6.7 (1.3–15.5)	.652
Coexistence of diabetes mellitus	0 (0%)	26 (6.5%)	.036
Regular user of anticholinergic drugs	5 (7.7%)	119 (29.8%)	** < .001**
Regular user of benzodiazepines	43 (66.2%)	289 (72.3%)	.305
Regular user of corticosteroids	1 (1.5%)	7 (1.8%)	1.000
Regular user of laxatives	56 (86.2%)	116 (29.0%)	** < .001**
Regular user of mood stabilizers	15 (23.1%)	48 (12.0%)	**.029**
Regular user of NSAID_S_	3 (4.6%)	36 (9.0%)	.335
Regular user of NaSSA	1 (1.5%)	9 (2.3%)	1.000
Regular user of SSRIs/SNRIs	3 (4.6%)	31 (7.8%)	.605
Regular user of TCA	0 (0%)	5 (1.3%)	1.000

Values are expressed as median (inter-quartile range) or number (percentage). Statistically significant values (*p* < .05) are given in bold

*CLZ* The clozapine exposure group, *non-CLZ* The clozapine non-exposure group, *SNRIs* Serotonin norepinephrine reuptake inhibitors, *SSRIs* Selective serotonin reuptake inhibitors, *NaSSA* Noradrenergic and specific serotonergic antidepressant, *NSAIDs* Non-steroidal anti-inflammatory drugs, *TCA* Tricyclic antidepressants

With regard to concomitant antipsychotics during clozapine administration, the top five drugs that were concomitantly administered for four or more weeks were quetiapine (23.1%), olanzapine (20.0%), risperidone (16.9%), levomepromazine (15.4%), and blonanserin (13.8%) (data not shown in Table 1).

### Patient characteristics of the appendicitis cases in both groups

The characteristics of all appendicitis cases in both the groups are summarized in Table 2. The age at the start of clozapine administration was 20.9 (15.9 − 26.7) years old, and the duration of clozapine administration were 16.7 (10.3 − 28.5) months.

**Table 2:** Patient characteristics of the appendicitis cases in both groups

**Characteristics**	**all cases**	**CLZ**	**non-CLZ**	***p*-value**
	*N* = 10	*n* = 5	*n* = 5	
Sex, Man	4 (40%)	2 (40%)	2 (40%)	1.000
Age at the onset of appendicitis (years old)	25.5 (20.8–38.8)	23.0 (16.7–28.6)	30.7 (24.1–59.9)	.117
Total daily dose of antipsychotics equivalent to chlorpromazine at the onset of appendicitis (mg)	770 (381–1172)	900 (750–1500)	425 (200–952)	.076
Age at the start of clozapine administration (years old)	-	20.9 (15.9–26.7)	-	-
Length of disease duration at the start of clozapine administration (years)	-	3.8 (1.3–8.4)	-	-
Duration of clozapine administration (months)	-	16.7 (10.3–28.5)	-	-
Maximum administration dose of clozapine (mg)	-	425 (250–600)	-	-
Dose of clozapine at the onset of appendicitis (mg)	-	400 (350–600)	-	-
Withdrawal of clozapine administration due to adverse events	-	0 (0%)	-	-
Age at the start of treatment for schizophrenia (years old)	18.4 (14.9–22.7)	16.1 (14.6–18.7)	21.6 (16.3–31.7)	.117
Age at the first visit to our hospital (years old)	21.5 (16.7–31.2)	19.1 (15.7–25.8)	26.1 (18.3–41.5)	.175
Length of the disease duration (years)	6.0 (4.3–17.6)	5.2 (2.2–10.8)	7.5 (4.8–32.0)	.251
Length of the observation period (years)	4.4 (1.8–8.7)	2.1 (1–3.8)	7.3 (4.4–18.5)	**.028**
Coexistence of diabetes mellitus	0 (0%)	0 (0%)	0 (0%)	-
Regular user of laxatives	7 (70%)	5 (100%)	2 (40%)	**.038**
Regular user of anticholinergic drugs	2 (20%)	0 (0%)	2 (40%)	.114
Regular user of benzodiazepines	9 (90%)	5 (100%)	4 (80%)	.292
Regular user of corticosteroids	1 (10%)	1 (20%)	0 (0%)	.292
Regular user of mood stabilizers	1 (10%)	0 (0%)	1 (20%)	.292
Regular user of NaSSA	1 (10%)	1 (20%)	0 (0%)	.292
Regular user of NSAID_S_	2 (20%)	1 (20%)	1 (20%)	1.000
Regular user of SSRIs/SNRIs	0 (0%)	0 (0%)	0 (0%)	-
Regular user of TCA	0 (0%)	0 (0%)	0 (0%)	-

Values are expressed as median (inter-quartile range) or number (percentage). Statistically significant values (*p* < .05) are given in bold

*CLZ* The clozapine exposure group, *non-CLZ* The clozapine non-exposure group, *SNRIs* Serotonin norepinephrine reuptake inhibitors, *SSRIs* Selective serotonin reuptake inhibitors, *NaSSA* Noradrenergic and specific serotonergic antidepressant, *NSAIDs* Non-steroidal anti-inflammatory drugs, *TCA* Tricyclic antidepressants

Regarding concomitant antipsychotics during clozapine administration, there were no drugs that were commonly used among all 5 patients with appendicitis in the clozapine exposure group (data not shown in Table 2).

### The cumulative incidences of appendicitis in both groups

**Fig. 1:**
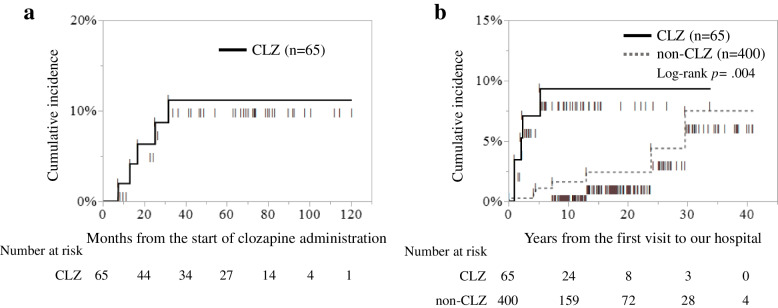
The cumulative incidences of appendicitis in both groups. CLZ, the clozapine exposure group; non-CLZ, the clozapine non-exposure group. Lines under the curve indicate censored cases; lines on the curve indicate appendicitis onset cases. **a** shows the cumulative incidence of appendicitis in the clozapine exposure group starting from the initiation of clozapine administration, and **b** shows the cumulative incidences of appendicitis in both groups starting from the initial visit. In the former, dropout was defined as withdrawal of clozapine administration or discontinuation of follow-up, and in the latter, dropout was defined as discontinuation of follow-up

To calculate the cumulative incidence of appendicitis in both groups, we used Kaplan–Meier analysis (Fig. 1). The last onset of appendicitis in the clozapine exposure group was observed approximately 32 months after the start of clozapine administration, after which the cumulative incidence plateaued at 11.2% (Fig. 1a). In contrast, Kaplan–Meier analysis showed that the cumulative incidence of appendicitis in the clozapine exposure group was significantly higher than that in the clozapine non-exposure group (log-rank *p* = 0.004; Fig. 1b). Approximately 30 years after the start of treatment for schizophrenia, the cumulative incidences reached plateaus of 9.4% and 7.5%, respectively (Fig. 1b).

### Multivariable analysis of the impact of clozapine on the onset of appendicitis

To further analyze the impact of clozapine on the onset of appendicitis, we used multiple logistic regression models (Table 3). In the univariate analyses, “the presence of a history of clozapine exposure” (*p* = 0.004), “age at the start of treatment for schizophrenia” (*p* = 0.034), and “regular use of laxatives” (*p* = 0.043) were all significant factors affecting the onset of appendicitis. The multivariate analysis was performed, including the following explanatory variables; “the presence of a history of clozapine exposure,” “age at the start of treatment for schizophrenia,” “regular user of corticosteroids,” and “regular user of NSAIDS,” according to model selection by AIC (χ^2^ = 16.015, df = 4, *p* = 0.003, McFadden's pseudo *R*^*2*^ = 0.166, AIC = 90.689). The multivariable analysis showed that only “the presence of a history of clozapine exposure” was an independent factor contributing to the onset of appendicitis (odds ratio = 6.458, 95% confidence interval = 1.674–24.915, *p* = 0.007). Given that the low incidence of appendicitis, this odds ratio means that the probability to develop appendicitis was approximately more than six times higher for patients treated with clozapine compared to those treated with other antipsychotics. Additionally, the observed coefficient of determination, the McFadden's pseudo R^2^, means to explain approximately one-sixth of the variance observed in this model.

**Table 3:** Multivariable analysis of the impact of clozapine on the onset of appendicitis

**Variables**		**Univariate**			**Multivariable**	
	**Odds**	**95% CI**	***p*-value**	**Odds**	**95% CI**	***p*-value**
Sex (Woman vs. Man)	1.056	0.294–3.794	.933	-	-	-
Presence of a history of clozapine exposure (Yes vs. No)	**6.583**	**1.851–23.419**	**.004**	**6.458**	**1.674–24.915**	**.007**
Age at the start of treatment for schizophrenia (years old)	**0.910**	**0.832–0.993**	**.034**	0.918	0.835–1.010	.078
Length of the disease duration (years)	0.966	0.908–1.029	.285	-	-	-
Coexistence of diabetes mellitus (Yes vs. No)	1.450 × 10^–6^	0– -	.990	-	-	-
Regular user of anticholinergic drugs (Yes vs. No)	0.682	0.143–3.258	.632	-	-	-
Regular user of benzodiazepines (Yes vs. No)	3.678	0.461–29.320	.219	-	-	-
Regular user of corticosteroids (Yes vs. No)	7.111	0.791–63.967	.080	9.739	0.863–109.853	.066
Regular user of mood stabilizers (Yes vs. No)	0.704	0.088–5.656	.742	-	-	-
Regular user of laxatives (Yes vs. No)	**4.101**	**1.046–16.073**	**.043**	-	-	-
Regular user of NaSSA (Yes vs. No)	5.506	0.629–48.167	.123	-	-	-
Regular user of NSAID_S_ (Yes vs. No)	2.824	0.579–13.788	.199	4.840	0.866–27.035	.072
Regular user of SSRI/SNRI (Yes vs. No)	1.423 × 10^–6^	0– -	.989	-	-	-
Regular user of TCA (Yes vs. No)	8.899 × 10^4^	0– -	.990	-	-	-

Statistically significant values (*p* < .05) are given in bold

*CLZ* The clozapine exposure group, *non-CLZ* The clozapine non-exposure group, *SNRIs* Serotonin norepinephrine reuptake inhibitors, *SSRIs* Selective serotonin reuptake inhibitors, *NaSSA* Noradrenergic and specific serotonergic antidepressant, *NSAIDs* Non-steroidal anti-inflammatory drugs, *TCA* Tricyclic antidepressants

## Discussion

In this retrospective study, we found that the incidence of appendicitis during clozapine exposure was prominently higher, approximately 20 times that of patients treated with other antipsychotics and the general population [7–9]. Additionally, we clarified, for the first time, that clozapine exposure is an independent factor contributing to the onset of appendicitis. Regarding the rationale for supporting our results, all cases of appendicitis in the clozapine-exposure group were observed during clozapine administration. Furthermore, none of the antipsychotics combined with clozapine that were commonly used by all patients with appendicitis in the clozapine exposure group were identified (data not shown). Additionally, patients with appendicitis in the clozapine-exposed group tended to have a shorter disease duration to appendicitis onset than those with appendicitis in the clozapine non-exposure group, although the difference was not significant (5.2 years vs. 7.5 years, *p* = 0.251). These findings support the hypothesis that clozapine exposure is associated with the onset of appendicitis in patients with schizophrenia, although the underlying pathological mechanism has not yet been elucidated.

Incidentally, the incidence of appendicitis during clozapine administration in our study was slightly lower than that reported in the report by Steinert [18]. One possible explanation for this is that approximately 30% of patients in the clozapine exposure group were transferred back to the referring hospital after the introduction of clozapine was completed; therefore, it was not possible to monitor the subsequent onset of appendicitis.

In addition, Steinert et al. reported that the mean age of appendicitis onset in six patients treated with clozapine was 34.4 years old, and the median duration of clozapine administration at the time of appendicitis onset was 26 (6–46) months [18]. Contrastingly, in our study, the median ages of appendicitis onset of the five patients treated with clozapine were 23.0 (16.7–28.6) years old, and the median duration of clozapine administration at the time of the appendicitis was 16.7 (10.3–28.5) months. Moreover, regarding patients with schizophrenia treated with clozapine, there was no marked difference in the incidence of appendicitis and the duration of clozapine exposure at the time of appendicitis onset between Steinert's report and ours (2,726 vs. 2,086 cases per 100,000 person-years, 26 months vs. 16.7 months, respectively). As described above, the incidence of appendicitis in all age groups is generally 90–110 cases per 100,000 persons per year, although it peaks in teens and then gradually declines. Specifically, there are no apparent differences in the incidence of appendicitis between age groups, except for teens [7–12]. Considering these facts, the impact of clozapine on the development of appendicitis may be roughly even across all ages.

The proposed mechanism of appendicitis is increased intraluminal pressure due to luminal obstruction, often by a fecalith, impacted stool, lymphoid hyperplasia, or malignancy, which leads to interference with circulation and ischemia of the appendiceal tissue, with consequent necrosis and bacterial invasion of the appendix [7–9, 23, 24]. Based on the above, two hypotheses can be considered for the mechanism by which clozapine causes appendicitis. Firstly, constipation is one of the typical side effects of antipsychotics, and is one of the major adverse events of clozapine [4, 5, 25]. A meta-analysis showed that clozapine is approximately three times more likely to cause constipation than other antipsychotics [26]. In contrast, a previous study found no obvious relationship between plasma clozapine concentration and constipation [26]. Consistent with previous research, the proportion of laxative users was significantly higher in the clozapine-exposed group than in the non-clozapine-exposed group (86.2% vs. 29.0%, *p* < 0.001). Steinert et al., who first reported the relationship between clozapine administration and appendicitis, also mentioned that two of six patients with appendicitis during clozapine administration exhibited subileus in the process of diagnosis [18]. Incidentally, constipation is a common side effect not only in clozapine users but also in other antipsychotic users. In fact, approximately 30% of the patients in the clozapine non-exposure group regularly used laxatives. However, the incidence of appendicitis in the non-clozapine exposure group was similar to that in the general population. Unfortunately, it cannot be further discussed in this study whether constipation is the main cause of appendicitis onset because the presence or absence of clinical constipation and its severity were not assessed in detail in each group.

Second, clozapine can cause inflammation, particularly myocarditis [27, 28]. Additionally, clozapine has been associated with other infections such as pneumonitis, hepatitis, pancreatitis, nephritis, and colitis [27, 28]. The mechanisms by which clozapine causes inflammation are not fully understood; nevertheless, it has been reported that pro-inflammatory cytokines, such as interleukin-6 (IL-6) and tumor necrosis factor-α (TNF-α), are elevated in patients with schizophrenia treated with clozapine [29, 30]. Several meta-analyses of cross-sectional studies have demonstrated that schizophrenia is related to the propensity to produce pro-inflammatory cytokines [31–33]. Considering these findings, it is possible that the underlying pro-inflammatory propensity of patients with schizophrenia, combined with the effects of clozapine, resulted in the development of appendicitis.

Finally, this study has several limitations. First, our results have insufficient validity due to the small sample size of the subjects in this study. Second, because of the retrospective nature of our study, demographic data, such as disease duration, were not comparable between the two groups. Furthermore, details of the exposure doses and exposure durations of drugs such as other antipsychotics, excluding clozapine, have not been investigated. In particular, patients with TRS were expected to have been exposed to higher chlorpromazine-equivalent doses of antipsychotics than non-TRS patients. Third, we did not identify the pathological mechanism by which clozapine caused appendicitis. Specifically, the presence or absence of clinical constipation and its severity were not evaluated in either group, pro-inflammatory cytokine concentrations such as IL-6 and TNF-α in both groups were not examined. Fourth, the plasma clozapine concentration at the onset of appendicitis was not measured in the clozapine exposure group, despite the fact that a few side effects are known to increase as clozapine concentration increases [34, 35]. Fifth, screening for a family history of appendicitis and inflammatory bowel disease was inadequate. The risk of appendicitis has been reported to be approximately three times higher in family members with a positive history of appendicitis than in those with no family history, despite no specific gene being identified [36]. Additionally, a previous study showed that loci had been identified that significantly mediate genetic correlations between schizophrenia and autoimmune diseases, such as ulcerative colitis and Crohn's disease [37]. Furthermore, Kooij et al. mentioned that the appendix may be the priming site for ulcerative colitis, focusing on previous reports that appendectomy in appendicitis was protective against the development of ulcerative colitis [38]. Sixth, our study was subject to bias. In Japan, a universal health insurance system has been adopted; thus, all citizens can receive necessary medical services anytime. However, as long as the medical history statement relies on the statements of the participants or their family members, its accuracy is limited. As described above, patients whose accuracy of their statements on medical history was questionable due to their schizophrenic medical condition and the absence of reliable relatives were excluded from this study, which leads to bias. It would be necessary to gain access to health insurance claims data for each patient to address this bias; however, the data were not available because of the retrospective nature of this study. Prospective studies are required to address these issues.

## Conclusions

Our data showed that the incidence of appendicitis in schizophrenic patients treated with clozapine was about 20 times higher than that in patients treated with other antipsychotics and the general population. Moreover, our results showed that clozapine exposure is a risk factor for appendicitis onset in patients with schizophrenia. These findings support the hypothesis that clozapine exposure is associated with the onset of appendicitis in patients with schizophrenia, which highlights the importance of psychiatrists prescribing clozapine to manage their patients’ conditions, with the risk of appendicitis in mind at all times.

## Data Availability

The datasets analyzed during the current study are not publicly available but are available from the corresponding author on reasonable request.

## Abbreviations

TRS: Treatment-resistant schizophrenia
DSM: Diagnostic and Statistical Manual of Mental Disorders
NaSSA: Noradrenergic and specific serotonergic antidepressants
SSRIs: Selective serotonin reuptake inhibitors
SNRIs: Serotonin norepinephrine reuptake inhibitors
TCA: Tricyclic antidepressants
NSAIDs: Non-steroidal anti-inflammatory drugs
IL-6: Interleukin-6
TNF-α: Tumor necrosis factor-α
